# Endometrial Carcinoma: A Comprehensive Analysis of Clinical Parameters, Treatment Modalities, and Prognostic Outcomes at a Tertiary Oncology Center in the UAE

**DOI:** 10.7759/cureus.48689

**Published:** 2023-11-12

**Authors:** Khalid S Balaraj, Nandan M Shanbhag, Abdulrahman Bin Sumaida, Syed Mansoor Hasnain, Omran A El-Koha, Rajmane Puratchipithan, Khalifa M Al Kaabi, Emad A Dawoud, Muhammad Y Nasim, Thikra A Hassan, Shilpi Roy

**Affiliations:** 1 Oncology/Radiation Oncology, Tawam Hospital, Al Ain, ARE; 2 Oncology/Palliative Care, Tawam Hospital, Al Ain, ARE; 3 Oncology/Internal Medicine, United Arab Emirtaes University, Al Ain, ARE; 4 Radiation Oncology, Tawam Hospital John Hopskins Medicine, Al-Ain, ARE; 5 Radiation Oncology, Tawam Hospital, Al Ain, ARE; 6 Oncology, Tawam Hospital, Al Ain, ARE; 7 Gynecologic Oncology, Tawam Hospital, Al Ain, ARE

**Keywords:** chemotherapy, adjuvant therapies, surgical intervention, treatment modalities, prognosis, microsatellite instability, pole ultramutated, molecular subtypes, uterine cancer, endometrial carcinoma

## Abstract

Background

Endometrial carcinoma (EC) remains a pressing global health issue, with a discernible upsurge in incidence, especially in developed countries. Notably, the United Arab Emirates (UAE) has witnessed a surge in EC cases, demanding an in-depth, region-specific exploration into the disease's clinical, treatment, and prognostic facets against the backdrop of its unique socio-genetic and environmental contours.

Aim

This study aimed to profess a comprehensive understanding of EC by examining clinical parameters, treatment modalities, and prognostic outcomes in the UAE context, thereby seeking to delineate potential correlations between varied therapeutic combinations, patient demographics, and tumor characteristics in affecting prognostic outcomes.

Materials and methods

A retrospective cohort study involving 93 patients diagnosed with EC from January 2011 to March 2023 at a leading oncology center in the UAE was conducted. Data, including demographic information, clinical presentation, treatment modalities, and prognostic outcomes, were meticulously extracted and analyzed. The R software (version 4.2.2) facilitated exhaustive statistical analyses, involving descriptive statistics, correlation analyses with the polycor package, and survival analyses utilizing the Kaplan-Meier method and Cox regression analysis via the survival and survminer packages, respectively.

Results

Although the correlation matrix revealed a noticeable relationship between “Family history” and “Age,” most parameters displayed independence, offering a robust platform for ensuing multivariate analyses. Kaplan-Meier survival curves, stratified by therapeutic modalities, exhibited no statistically significant survival differences across therapeutic cohorts (p-values: 0.44, 0.86, and 0.83). Conversely, the composite Cox regression model underscored “non-national” demographic, Diabetes Mellitus II, and stromal invasion as pivotal prognostic factors, indicating the multifactorial nature of survival in EC patients and emphasizing demographic and tumor characteristics over therapeutic modalities as influential prognostic determinants.

Conclusion

In conclusion, while therapy types were not directly correlated with survival, demographic and tumor traits prominently impacted prognostic outcomes, advocating for an intricate, multidimensional approach to managing EC in the UAE. This study hopes to sow seeds for subsequent research, shaping clinically and culturally apt practices and policies in the region’s healthcare landscape.

## Introduction

Endometrial carcinoma is a significant health concern with a growing global incidence [1]. The disease's molecular intricacies have led to its categorization into distinct subtypes, each with its prognostic implications [2]. As the most common gynecological malignancy in developed nations, its increasing prevalence is attributed to various risk factors, including obesity, hypertension (HTN), and diabetes [3].

The United Arab Emirates (UAE), with its rapid socio-economic advancements, has experienced shifts in lifestyle patterns and healthcare infrastructure. Such transitions, while beneficial in many respects, have also introduced challenges, particularly in the realm of non-communicable diseases, including cancers. Recent data suggests an escalating incidence of endometrial carcinoma in the UAE, emphasizing the need for a region-specific understanding of the disease's clinical parameters, treatment modalities, and prognostic outcomes [4].

The European consensus on endometrial carcinoma has provided multi-disciplinary evidence-based guidelines, aiming to enhance the quality of care for affected women globally. These guidelines encompass a comprehensive approach, from staging and molecular markers to management strategies for various disease stages [5]. However, the unique demographic and healthcare landscape of the UAE necessitates a tailored approach to managing endometrial carcinoma, ensuring optimal patient outcomes.

Prognostic outcomes for endometrial carcinoma are multifaceted. While the disease stage at diagnosis remains a primary determinant of survival, molecular markers, histological grade, and other clinical parameters play a pivotal role in influencing outcomes [6,7]. In the UAE's context, understanding these factors is crucial, given the region's distinct genetic, cultural, and environmental attributes.

Molecular characterization has revolutionized our understanding of endometrial carcinoma. Recent studies have identified four primary molecular subgroups: POLE ultra-mutated, microsatellite instability hypermutated, copy-number low, and copy-number high. Each subgroup presents unique clinical behaviors and responses to treatment. For instance, the POLE ultra-mutated subtype, while rare, is associated with a favorable prognosis, whereas tumors with p53 mutations often exhibit aggressive behavior and poor outcomes [8]. Such molecular stratifications not only aid in predicting disease progression but also in tailoring treatment modalities to ensure maximum therapeutic efficacy.

Treatment modalities for endometrial carcinoma are diverse and often tailored to the disease's stage and molecular profile. Surgical intervention remains the cornerstone of treatment, especially for early-stage tumors [9]. However, advanced stages and specific high-risk molecular subtypes may necessitate adjuvant therapies, including chemotherapy, radiotherapy, and targeted biological therapies [10]. The advent of proteogenomics has further enriched our understanding, revealing potential pathways and offering new avenues for targeted therapies [11]. In the context of the UAE, where healthcare infrastructure is rapidly advancing, integrating these state-of-the-art treatment modalities can significantly enhance patient outcomes.

Understanding regional variations in endometrial carcinoma is of paramount importance. With its unique genetic pool influenced by consanguinity, the UAE may present distinct molecular patterns of the disease. Additionally, lifestyle factors, including dietary habits, physical activity levels, and cultural practices, can influence disease epidemiology and outcomes. Recognizing these regional nuances is crucial for developing effective screening programs, public health campaigns, and personalized treatment strategies.

This study presents a comprehensive analysis of endometrial carcinoma's clinical parameters, treatment modalities, and prognostic outcomes at a leading oncology center in the UAE. As the UAE strides towards becoming a global healthcare hub, research endeavors like this study are pivotal in ensuring that care paradigms are evidence-based, culturally sensitive, and tailored to the region's unique needs. Through this study, we aim to provide valuable insights that could shape future clinical practices and research in the region.

## Materials and methods

Study design and setting

A retrospective cohort study was undertaken at a premier oncology center in the UAE. The primary objective was to conduct an exhaustive analysis of the clinical parameters, treatment approaches, and prognostic outcomes in patients diagnosed with endometrial carcinoma from January 2011 to March 2023.

Patient selection

Inclusion criteria encompassed patients with a histopathologically confirmed diagnosis of endometrial carcinoma. Patients diagnosed with other simultaneous malignancies or incomplete medical documentation or who underwent their primary treatment outside the study centre were excluded from the study.

Data collection

A meticulous review of medical records was executed to extract (Table 1).

**Table 1:** Descriptive statistics summary for endometrial cancer patient cohort (N=93). Please note all the percentages are rounded to the nearest whole number for ease of reading the data only. EBRT - External Beam Radiotherapy; HDR-Brachy - High-Dose Rate Brachytherapy; SD - Standard Deviation

	Mean	SD	min	25%	50%	75%	max
Age	60.36	12.32	26	54	61	67	98
Days between 1st visit to start of Radiotherapy	24.23	10.78	10	18	21	28	78
Overall Treatment time in days (Radiotherapy)	46.87	7.96	16	43	46	51	75
Weight loss in kgs (Before Treatment -After Treatment)	10.08	12.59					48
Nationality	National (%)	Non-National (%)					
	23 (24)	70 (75)					
Histopathological Features	Yes (%)	No (%)					
Lymphovascular Invasion	35 (37)	58 (62)					
Stromal Invasion	28 (30)	65 (69)					
Myometrial Invasion	46 (49)	47 (50)					
Lower Uterine Involvement	40 (43)	53 (56)					
Lymph nodes	14 (15)	79 (84)					
Grade	I (%)	II (%)	III (%)				
	24 (25)	17 (18)	44 (47)				
Treatment	Yes (%)	No (%)					
Chemotherapy	48 (51)	45 (49)					
Surgery	93 (100)						
EBRT	93 (100)						
Dose in Gy (EBRT)	45						
Number of Fractions (EBRT)	25						
HDR-Brachy	76 (82)	17 (18)					
Dose in Gy (HDR-Brachy)	10						
Number of Fractions (HDR-Brachy)	2						
Treatment (Completed)	89 (95)						
Survival	Yes (%)	No (%)					
Alive	79 (84)	14 (15)					
Progression	47 (50)	46 (50)					
Co-Morbidities	Yes (%)	No (%)					
Diabetes Mellitus II	26 (28)	57 (72)					
Hypertension	27 (29)	56 (71)					
Dyslipidemia	22 (24)	71 (76)					
Family history of cancer	15 (16)	78 (84)					

Demographic information - age at diagnosis, nationality, and associated comorbidities. Clinical presentation - initial symptoms and their duration. Clinical parameters - tumor stage, grade, histological subtype, and molecular characterization. Treatment modalities - surgical procedures, chemotherapy protocols, details of radiotherapy, and any administered targeted or hormonal therapies. Prognostic outcomes - recurrence of the disease, metastatic events, date of death and date of last review.

Statistical analysis

Data were analyzed using R version 4.2.2. Descriptive statistics summarized the data, with categorical variables presented as frequencies and percentages. Continuous variables were delineated as means ± standard deviations or medians accompanied by interquartile ranges, contingent on the data distribution. The polycor package (version 0.8) in R was employed for correlations involving mixed variables (numeric and categorical). Survival analysis was executed using the Kaplan-Meier method, with the survival package (version 3.5) in R. Differences in survival were gauged using the log-rank test. The multivariate Cox regression analysis, facilitated by the survminer package (version 0.4.9) in R, identified independent prognostic determinants. A p-value <0.05 was deemed statistically significant.

Ethical considerations

The Institutional Review Board (IRB) of the oncology center approved this study with IRB approval number MF2058-2021-795. Owing to its retrospective design, the requirement for informed consent was exempted. Rigorous measures ensured the confidentiality of patient data, with all records de-identified prior to analysis.

## Results

Patient characteristics

In our comprehensive investigation into endometrial carcinoma, we meticulously analyzed a cohort comprising 93 patients. The primary focus was discerning the ramifications of various therapeutic combinations on patient prognoses. Patients were stratified based on the amalgamation of treatment modalities: particularly HDR-Brachy and Chemotherapy (Brachy-NO: Chemo-NO, Brachy-NO: Chemo-YES, Brachy-YES: Chemo-NO, and Brachy-YES: Chemo-YES). The distribution across these stratifications was 9, 8, 36, and 40 patients.

The dataset encompasses a diverse age group of endometrial cancer patients, from the youngest at 26 years to the eldest at 98 years with a mean of 60.36 years. This mean value underscores the median age range in which endometrial cancer is most commonly diagnosed or observed in this particular dataset (Table 1).

Correlation analysis

**Figure 1:**
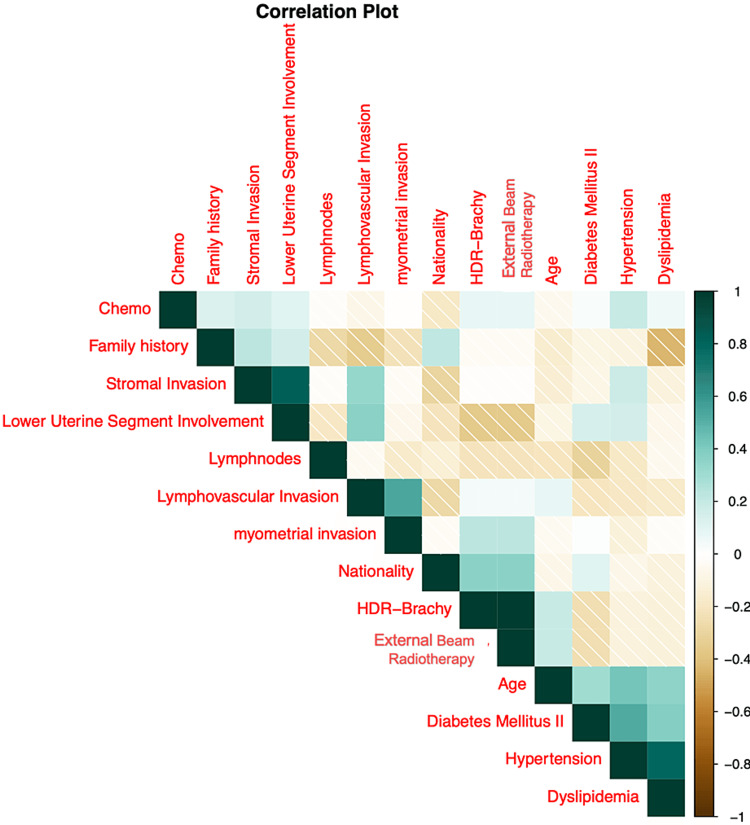
Correlation plot HDR_brachy: High-Dose Rate Brachytherapy; chemo: chemotherapy

To elucidate potential interrelationships between diverse clinical and demographic parameters, we employed a correlation matrix, graphically represented in Figure 1. This matrix unveils the intricate dynamics between the parameters, elucidating potential synergistic or antagonistic interactions.

A discernible correlation was observed between “Family history” and “Age,” underscoring the potential genetic and environmental interplay. A familial predisposition to malignancies was discernible across the cohorts, with the Brachy-NO: Chemo-YES cohort manifesting the most pronounced predisposition at 37.5%. The mean age at diagnosis spanned from 55.33 years (SD 22.74) in the Brachy-NO: Chemo-NO cohort to 62.31 years (SD 11.26) in the Brachy-YES: Chemo-NO cohort. Statistical analysis revealed no significant age-related variance across the cohorts (p=0.405).

A salient proportion of patients across each cohort were diagnosed with comorbidities, notably diabetes mellitus type II (DMII) and HTN. The Brachy-NO: Chemo-YES cohort exhibited the highest prevalence at 62.5% and 50%, respectively. Parameters such as lymph node involvement, lymphovascular invasion, stromal invasion, and myometrial invasion were uniformly distributed across the cohorts, with no statistically significant disparities (Table 2).

**Table 2:** Patient groups and characteristics SD: Standard Deviation

	Brachy-NO:Chemo-NO	Brachy-NO:Chemo-YES	Brachy-YES:Chemo-NO	Brachy-YES:Chemo-YES	P-value
n	9	8	36	40	
Family history = YES (%)	0 ( 0.0)	3 ( 37.5)	6 ( 16.7)	6 ( 15.0)	0.21
Age (mean (SD))	55.33 (22.74)	57.12 (12.61)	62.31 (11.26)	60.40 (9.91)	0.41
Nationality = non-national (%)	5 (55.6)	5 ( 62.5)	32 ( 88.9)	30 ( 75.0)	0.097·
Diabetes Mellitus II = YES (%)	4 (44.4)	5 ( 62.5)	13 ( 36.1)	14 ( 35.0)	0.50
Hypertension = YES (%)	4 (44.4)	4 ( 50.0)	11 ( 30.6)	18 ( 45.0)	0.54
Dyslipidemia = YES (%)	3 (33.3)	2 ( 25.0)	7 ( 19.4)	10 ( 25.0)	0.83
Lymph nodes = Positive (%)	3 (33.3)	1 ( 12.5)	4 ( 11.1)	6 ( 15.0)	0.42
Lymphovascular Invasion = YES (%)	3 (37.5)	2 ( 25.0)	12 ( 35.3)	12 ( 32.4)	0.94
Stromal Invasion = YES (%)	1 (12.5)	3 ( 37.5)	8 ( 23.5)	10 ( 27.0)	0.70
myometrial invasion = >HALF (%)	4 (66.7)	2 ( 25.0)	18 ( 54.5)	22 ( 62.9)	0.25
Lower Uterine Segment Involvement = YES (%)	2 (28.6)	7 ( 87.5)	12 ( 35.3)	11 ( 30.6)	0.023*
p-values: · p-val<0.1, *p-val<0.05, **p-val<0.01, *** pval<0.001					

However, a marked difference was evident in the prevalence of lower uterine segment involvement across the cohorts (p=0.023), with the Brachy-NO: Chemo-YES cohort registering the highest prevalence at 87.5%. This granular analysis of patient demographics and clinical characteristics furnishes a robust foundation for subsequent evaluations of therapeutic efficacy and prognostic outcomes.

Despite these correlations, the majority of the parameters exhibited independence, bolstering the robustness of our subsequent multivariate analyses. This independence ensures an unbiased evaluation of each parameter's contribution to the prognostic model.

Survival analysis

**Figure 2:**
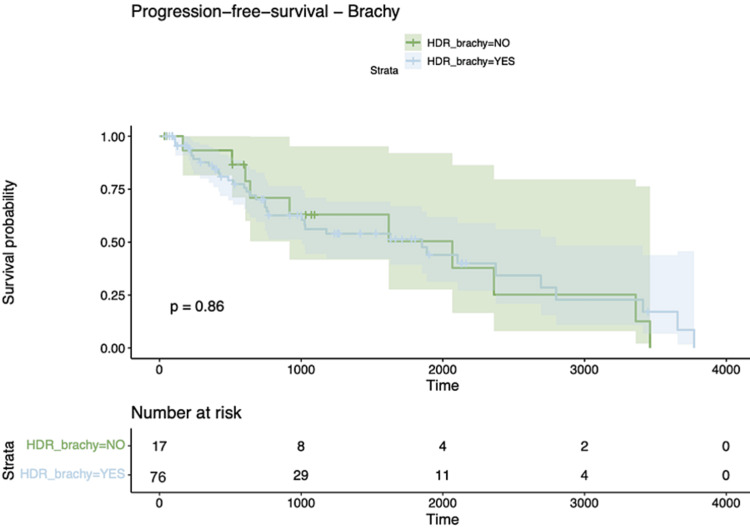
Kaplan Meier curve for brachytherapy for PFS PFS: Progression-Free Survival; HDR_brachy: High-Dose Rate Brachytherapy

**Figure 3:**
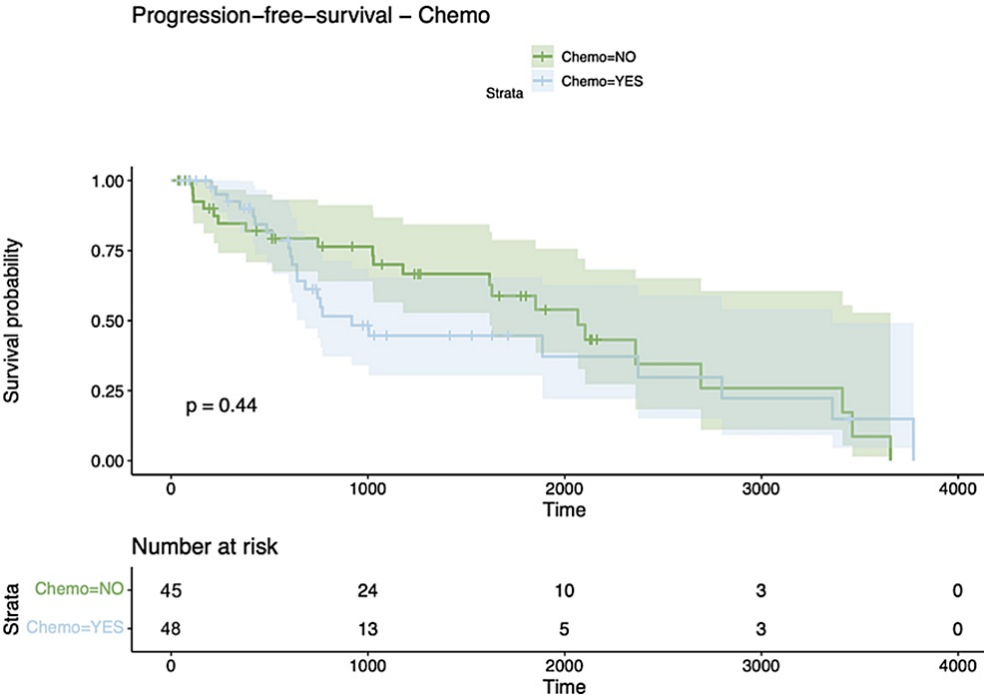
Kaplan Meier curve for chemotherapy group for PFS PFS: Progression-free survival

**Figure 4:**
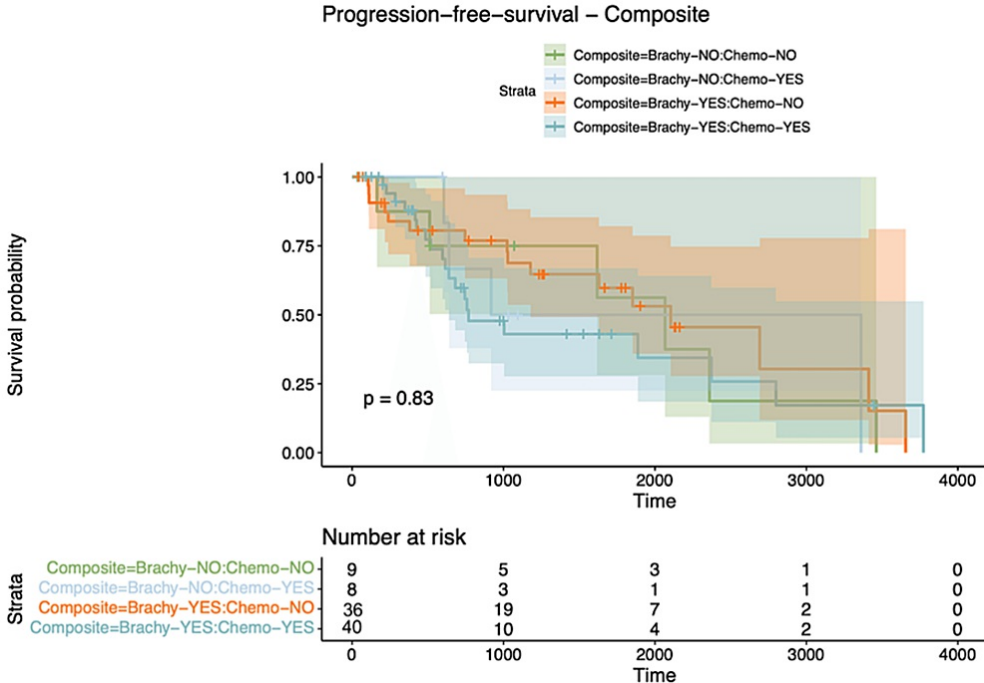
Kaplan Meier curve for the composite group for PFS PFS: Progression-free Survival

After the correlation analysis, we embarked on a survival analysis. Kaplan-Meier survival curves stratified by therapeutic modalities representing Chemotherapy, HDR-Brachytherapy, and Composite therapeutic modalities all yielded null results, with p-values of 0.44, 0.86, and 0.83, respectively, signifying no statistically significant survival disparities across the therapeutic cohorts (Figures 2-4).

**Figure 5:**
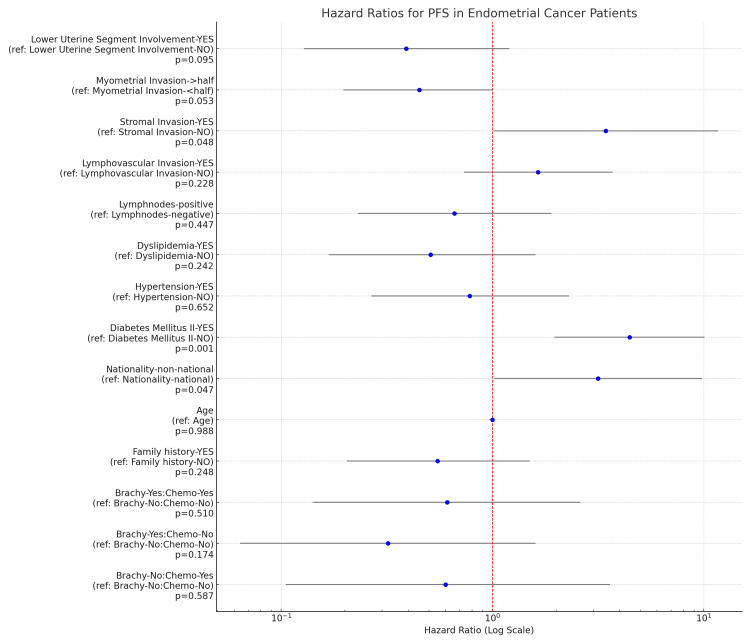
Hazard ratios for the PFS PFS: Progression-free Survival

However, a composite Cox regression model revealed pronounced disparities in patient demographics about progression-free survival. Specifically, the “non-national” demographic emerged as a significant prognostic factor, with a hazard ratio (HR, 95% CI) of 3.16, 1.02-9.80 (p < 0.047) (Figure 5).

Additionally, the presence of DMII and stromal invasion were identified as significant prognostic determinants, emphasizing the multifactorial nature of survival in endometrial carcinoma patients. In summation, while therapeutic modalities did not manifest a significant impact on survival, our analysis accentuates the importance of patient demographics and tumor characteristics in prognostic evaluations, underscoring the multifaceted determinants of survival in endometrial carcinoma patients.

## Discussion

Endometrial cancer, one of the most common gynecological malignancies, has several prognostic factors that influence survival outcomes. Among these, lymphovascular space invasion (LVSI) has emerged as a significant predictor of both lymph node metastasis and overall survival. A study conducted in early-stage endometrial cancer patients found that LVSI significantly and independently influenced the three-year and five-year survival rates [12]. Another study echoed these findings, emphasizing that patients with lower International Federation of Obstetrics and Gynecology (FIGO) stages but with confirmed LVSI presence may be at an increased risk of recurrence and poor overall survival [13].

Myometrial invasion, another pivotal factor, has been linked to positive LVSI, lymph node metastasis, cancer recurrence, and poor overall survival for endometrial cancer patients [14]. This suggests that the depth of tumor invasion into the myometrium is a crucial determinant of prognosis. Furthermore, the involvement of the lower uterine segment has been associated with poorer outcomes in endometrial cancer patients, emphasizing the importance of tumor location in prognostication [15].

Tumor grade and FIGO stage are well-established prognostic factors in endometrial cancer. Higher tumor grades and advanced FIGO stages are generally associated with a worse prognosis, underscoring the importance of early detection and intervention [16].

The impact of comorbidities, particularly DMII and HTN, on the prognosis and survival of endometrial cancer patients has been a subject of interest in oncological research. These comorbidities, often prevalent in the general population, can have profound implications on the management and outcomes of endometrial cancer.

Diabetes mellitus, specifically type II, has been linked to a range of malignancies, including endometrial cancer. A prospective database analysis highlighted that type 2 diabetes mellitus (T2DM) confers an increased risk of death in endometrial cancer patients [17]. This association underscores the importance of vigilant monitoring and management of diabetic endometrial cancer patients. Another study emphasized that diabetes mellitus plays a pivotal role in the development of premenopausal endometrial cancer, especially in patients with a BMI above 30 kg/m^2 ^[18]. The intricate relationship between diabetes and endometrial cancer is further complicated by factors such as obesity, insulin resistance, and hyperinsulinemia, which can influence the tumor microenvironment and potentially drive cancer progression.

HTN is another common comorbidity that has been investigated in the context of endometrial cancer. A study examining the impact of comorbid conditions on survival in endometrial cancer found that attention to comorbid conditions, especially DM and HTN, is becoming increasingly relevant given the favorable survival rate for most patients with endometrial cancer [19]. Another study highlighted that hypertensive black women experienced better survival outcomes, suggesting a potential interplay between HTN, race, and endometrial cancer prognosis [20]. However, the exact mechanisms underlying this relationship remain elusive and warrant further investigation.

It is worth noting that both DMII and HTN are components of the metabolic syndrome, which has been associated with an increased risk of endometrial cancer [21]. The presence of these comorbidities can influence treatment decisions, response to therapy, and overall prognosis. For instance, patients with these comorbidities might be at higher risk of postoperative complications, which can affect their overall treatment plan [22].

Age is a significant factor in the onset and progression of endometrial cancer. A study supports the hypothesis that late menarcheal age is inversely associated with endometrial cancer risk, emphasizing the intertwined nature of age and hormonal influences in the disease's development [23]. Furthermore, another study reported that the incidence rates for endometrial cancer have been on the rise, highlighting the importance of age as a crucial factor in understanding the disease's epidemiology [24]. Women who bear children later in their reproductive life may have a reduced risk for endometrial cancer compared to those who complete their families earlier [25]. Another study found that obesity is linked to an earlier age at diagnosis of endometrioid-type endometrial cancers, emphasizing the complex interplay between age, obesity, and the risk of developing this malignancy [26].

The impact of various treatment modalities on the survival of endometrial cancer patients has been known. Surgical intervention remains the cornerstone of endometrial cancer treatment. A study analyzing the time to surgery in endometrial cancer patients found that an increased time to surgery was negatively associated with overall survival in early-stage type I endometrial cancer [27]. Another study emphasized the importance of surgical staging, revealing that lymph node staging for tailoring adjuvant therapy led to improved survival outcomes in non-endometrioid endometrial cancer [28]. Furthermore, the surgical approach, whether minimally invasive or open, can influence survival outcomes. A study comparing minimally invasive surgery to open surgery in stage II endometrial cancer patients found less favorable survival outcomes with the minimally invasive approach [29].

Chemotherapy, often used in combination with other treatments, can be beneficial, especially in advanced or recurrent endometrial cancer. However, the specific impact of chemotherapy on survival varies based on the stage and type of endometrial cancer. While some studies have shown a survival benefit with the use of adjuvant chemotherapy, others have not found a significant difference [30].

Radiotherapy plays a pivotal role in the management of endometrial cancer, especially in advanced stages or high-risk cases. A study focusing on women with high-grade early-stage endometrial cancer treated with postoperative adjuvant radiotherapy found that tumor stage and grade were independent predictors for overall survival [31]. Another study highlighted that treatment with multi-agent chemotherapy followed by radiotherapy was associated with longer overall survival compared to radiotherapy followed by chemotherapy or treatment alone [32].

Brachytherapy, a form of internal radiation therapy, has been employed as an adjuvant treatment for endometrial cancer. A study by Sorbe et al. (2012) demonstrated that adjuvant vaginal brachytherapy significantly reduced local recurrences in intermediate-risk endometrial cancer patients without affecting overall survival [33].

Limitations

Retrospective Design

The study's retrospective nature can introduce biases related to patient selection and data recording. The reliance on historical data might also limit the availability and quality of pertinent information.

Limited Sample Size

The utilization of a relatively small cohort of 93 patients might restrict the statistical power of the study and the generalizability of the findings to a broader population.

Single-Center Perspective

Being conducted at a single oncology center, the study might not capture the variability and nuances present in different healthcare settings, thus potentially limiting the applicability of its findings across the UAE or other regions.

Variability in Treatment and Management

There may be inconsistencies or evolutions in treatment protocols over the study period (2011-2023), which might introduce variables that could affect the validity and reliability of the associations and outcomes observed.

Lack of Comprehensive Genetic and Lifestyle Data

The potentially insufficient availability of detailed genetic and lifestyle-related information may limit the ability to deeply explore and understand the specific risk factors, treatment responses, and prognostic outcomes among different patient subsets.

## Conclusions

In conclusion, our study on a cohort of 93 patients from the UAE provides insights into the multifactorial determinants that influence endometrial carcinoma outcomes. While therapeutic modalities such as Brachytherapy and Chemotherapy play a vital role, patient demographics, tumor characteristics, genetic predispositions, and comorbid conditions like DMII are equally significant. Particularly notable is the influence of nationality and the profound impact of familial history of malignancies, underscoring the need for genetic counseling and targeted screenings. Moreover, individualized treatment plans, factoring in tumor stage, grade, and histological subtype, remain paramount for optimizing survival outcomes. The intricacies of these determinants not only steer clinical decision-making but also shed light on the disease's biology, heralding a path for future targeted therapeutic strategies.
